# Editorial: A new age for articular cartilage: from bench to beside of tissue engineering and regenerative medicine in cartilage tissue repair

**DOI:** 10.3389/fbioe.2024.1519455

**Published:** 2024-11-29

**Authors:** Piergiorgio Gentile, Valentina Giudice, Erwin Pavel Lamparelli

**Affiliations:** ^1^ Centre for Biomaterials and Tissue Engineering (CBIT), Universitat Politècnica de València, València, Spain; ^2^ Hematology and Transplant Center, University Hospital “San Giovanni di Dio e Ruggi d’Aragona”, Salerno, Italy; ^3^ Department of Medicine and Surgery, University of Salerno, Baronissi, Italy

**Keywords:** articular cartilage, tissue engineering, regenerative medicine, tissue repair, personalized medicine

## 1 Introduction

Articular cartilage is a key part of the synovial joints, which also include the synovial cavity and joint capsule. Various events can cause articular cartilage lesions, such as traumas, sports-related activities, occupational accidents, or autoimmune diseases. These can lead to chronic conditions known as osteoarthritis (OA) because cartilage has a limited blood supply and low metabolic activity, preventing it from healing on its own (Richmond et al., 2013). Detailly, OA is characterized by structural deterioration of hyaline cartilage with subsequent exposure of underlying bones (Man and Mologhianu, 2014). Current clinical management of articular cartilage injuries and OA represents a significant challenge in orthopedics. Available treatments are largely conservative (e.g., pharmacotherapy, arthrocentesis, and physiotherapy) or surgical, primarily focusing on relieving pain and inflammatory symptoms to decrease functional disability and complications (Żylińska et al., 2018). However, no existing medications can completely heal or regenerate cartilage lesions.

**FIGURE 1:**
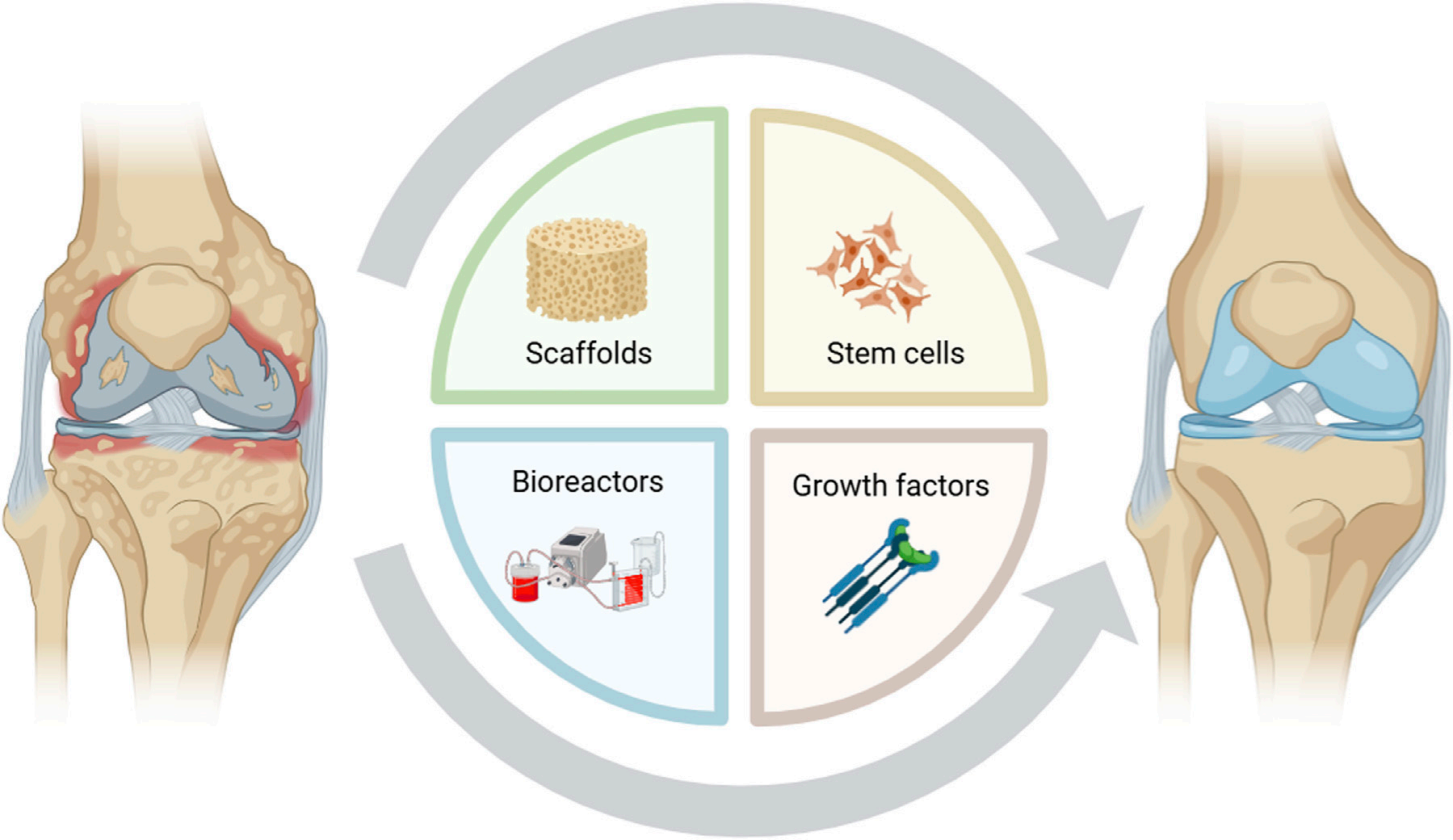
Representation of main tools used in tissue engineering protocols.

Tissue engineering is an innovative and promising therapeutic approach in which cells, scaffolds, and chemical or mechanical signals are organized to mimic biological tissues. Once optimized *in vitro*, synthetic tissues can be grafted to damaged sites to guide regeneration (Figure 1). Interactive and bioengineered supports, also termed 3-dimensional (3D) scaffolds, are implanted to stimulate new cartilage formation and promote stem cell differentiation toward a chondrogenic phenotype. 3D scaffolds, composed of biocompatible and biodegradable polymers, can host stem cells and growth factors to generate a bioengineered tissue mimicking physiological conditions; however, biomaterials must comply with strict requirements to be adopted in clinical settings, including biocompatibility, biodegradability, and low immunogenicity (Song et al., 2018). Moreover, these biomaterials should exhibit suitable mechanical properties to avoid structural degradation once implanted *in vivo*. Therefore, the proper choice of biomaterials and human stem cells, as well as chemical and mechanical stimuli, is critical to the success of tissue engineering (Lamparelli et al., 2022).

In this research topic of *Frontiers in Bioengineering and Biotechnology*, we gathered original works and reviews that provide state-of-the-art about cartilage tissue engineering, focusing on recent 3D *in vitro* models, including organoids under static or dynamic conditions or employing innovative scaffold-based or high-density cultures. Furthermore, we included bibliometric analysis for exploring research interest in OA joint distraction treatments and original articles on the biological significance of microRNAs in mitigating inflammation and fibrosis in 3D *in vitro* OA models.

In their review, Zeng et al. have summarized advancements in new OA models using cartilage organoids, 3D structures that mimic cartilage architecture and physiological functions. These models are beneficial for disease modeling, drug screening, and regenerative medicine purposes. However, cartilage organoids still face many challenges before their application in clinical research and regenerative medicine because they poorly reproduce *in vivo* functions, distribute nutrients, growth factors, and oxygen diffusion at the core of 3D masses, and ensure proper interactions between different cell types (Zeng et al.).

In their original article, Peng et al. conducted a bibliometric analysis of joint distraction within OA treatment research, examining over 450 scholarly articles, mostly from the United States, as leaders in international collaboration, publication count, and citation frequency. However, most studies focused on nonsurgical interventions and joint arthroscopy procedures, with limited research on joint distraction for OA treatment (Peng et al.).

The work by Pfeifer et al. explores the expression of microRNA-140 in equine synovial-membrane-derived mesenchymal stem cells-derived extracellular vesicles under inflammatory conditions in 2D- and 3D-OA *in vitro* models, as this microRNA has anti-inflammatory and anti-fibrotic properties and is produced in response to inflammation to quickly restore homeostasis, especially under 3D conditions (Pfeifer et al.).

The review by Cui et al. deals with common osteochondral staining methods, criteria for high-quality histological images, and current histological scoring systems for osteochondral regeneration with tissue-engineered grafts in synovial joint pathological degeneration. They emphasize the importance of assessing the cartilage layer, new bone formation, and graft–host interface through histological staining, and suggest including the subchondral bone plate in the scoring system for osteochondral regeneration (Cui et al.).

Finally, Cheng et al. use a 3D printing technology to fabricate a triphasic scaffold of PLA/PCL-PLGA/Mg(OH)₂, composed of a cartilage layer, an osteochondral interface, and a bone layer, and filled with Velvet antler polypeptides, a bioactive peptide, for promoting *in vitro* osteogenesis and chondrogenesis of fibrocartilage stem cells. This biocompatible scaffold could represent a promising tool for bone and cartilage tissue engineering in osteochondral defects treatment, likely due to its hierarchical structure that creates distinct microenvironments for cartilage and bone tissues (Cheng et al.).
